# Employees Diagnosed with Cancer: Current Perspectives and Future Directions from an Employer’s Point of View

**DOI:** 10.1007/s10926-018-9802-x

**Published:** 2018-07-23

**Authors:** Sietske J. Tamminga, Marije D. J. Wolvers, Michiel A. Greidanus, AnneClaire G. N. M. Zaman, Anna M. Braspenning, Monique H. W. Frings-Dresen, Angela G. E. M. de Boer

**Affiliations:** Coronel Institute of Occupational Health, Academic Medical Center (AMC), University of Amsterdam, Amsterdam Public Health Research Institute, Amsterdam, The Netherlands

**Keywords:** Neoplasm, Employer, Return to work, Intervention

## Abstract

*Purpose and methods* Cancer survivors have a higher risk of adverse work outcomes such as not being able to return to work (RTW). The process of returning to work is complex as a result of the diverse stakeholders and numerous factors involved related to the employee diagnosed with cancer, the work environment, health care system, and the legal system. One of the key stakeholders is the employer, as the employer is in the position to facilitate work accommodations. Therefore, the purpose of this brief review is to consider opportunities regarding the role of the employer to enhance the work participation of employees with cancer. *Results and conclusions* We currently know little about which aspects of employer support have a positive impact on the ability of employees diagnosed with cancer to retain at work or RTW. In addition, there is a lack of interventions and tools which support employers in their management of employees diagnosed with cancer. The inclusion of employer support into the workplace can help employees diagnosed with cancer with their work retention and RTW, which is an important aspect of their quality of life and benefits the society at large.

Increasing survival rates of cancer have strengthened the focus on survivorship care. Survivorship care not only includes treatment of medical late effects and after care, but also incorporates quality of life, psychological, and societal outcomes [1]. In this light, the founding editor of this journal Prof. Dr. Michael Feuerstein appealed in 2005 to extend our research field to the labour participation of employees diagnosed with cancer [2]. Thirteen years later, an increasing number of papers addressing this important issue have been published both from an occupational health perspective [3] as from an oncological perspective [4]. From this research we have learned that, as is the case in other populations, work provides structure, social contacts, distraction, and financial security, and can thereby contribute to a better quality of life of employees diagnosed with cancer [5, 6]. However, resuming work during or after cancer treatment can be challenging [7]. The process of returning to work is complex as a result of the diverse stakeholders and numerous factors involved related to the employee diagnosed with cancer, the work environment, health care system, and the legal system [7–9]. Additionally, employees may be confronted with specific cancer-related issues such as the unpredictable course of the disease, disabling treatments, stigma [10, 11], discrimination [12], fearful attitudes by line managers [13], and re-evaluation of the importance of work [14].

Factors related to the work environment have shown to impact work outcomes. These factors include support by supervisors and colleagues, flexibility, atmosphere at work, work accommodations, and work stress [7] which can all be influenced by the employer. Employers are in the position to support employees diagnosed with cancer and create facilitating conditions for them to participate at work [12]. Research further found that female employees diagnosed with breast cancer who work for accommodating employers are more likely to retain their jobs after treatment [15]. Moreover, employees diagnosed with cancer identified employers as one of the main stakeholders to facilitate their attempt to retain at work or return to work (RTW) [16, 17]. However, knowledge about the role of the employer during RTW of employees diagnosed with cancer is predominantly gathered from qualitative studies among employees diagnosed with cancer, while few qualitative studies have been performed from the perspectives of employers [12, 18]. In these few qualitative studies, employers mentioned to be uncertain about how and when to communicate and therefore avoided cancer-related discussions [19]. Also, employers indicated that guiding an employee diagnosed with cancer is complicated and demanding, among others because of privacy issues and a lack of cooperation with primary care [20, 21]. Moreover, these studies indicated that employers need supportive information and tools [20, 22]. However, work support interventions are mainly delivered in a clinical setting, while involving the employer appeared to be difficult [10], therefore, it might be efficient to start with studying how, what, and why (not) work support interventions are currently integrated in the care process, in order to explore barriers and success factors of similar interventions. To the best of our knowledge, no intervention studies on the role of the employer in their management of an employee diagnosed with cancer have been published in the scientific literature. To enhance and sustain work participation of employees diagnosed with cancer, the focus of future research should therefore be widened to better capture the role of the employer and provide them with tools and interventions. When developing such tools and interventions for employers, we could learn from the grey literature in which several interventions have been described (mainly for larger companies), with ‘MacMillan Cancer Support’ as one of the most comprehensive and well known examples [23]. Although a recent study found that breast cancer survivors in developed and developing countries experience the same work-related challenges [24], implementation of an intervention could be affected by differences between a countries’ legislation and healthcare system. That is for instance, which stakeholders are involved and to what degree and which of their behaviour is incentivised [25]. Considering the above, we propose that the following four components should be studied and addressed in more detail: (1) communication between employer and employee diagnosed with cancer; (2) attention for small and medium enterprises (SMEs); (3) close collaboration between all stakeholders; and (4) implementation of interventions involving the employer. First, communication should start early after initial diagnosis in order to achieve a satisfactory, timely, and open dialogue between the employee diagnosed with cancer and his or her employer [20]. In case of an employee diagnosed with cancer, this is particularly important to prevent misconceptions about preferences and possibilities regarding when and how to keep in touch and the employee’s participation at work [11]. Second, little is known about SMEs with regard to their possibilities to provide support for RTW [26, 27]. We do know that SMEs may lack possibilities to manage employees with work restrictions or on sick leave, for example by offering modified work or structural occupational (health) care [26]. Also, they may not have experience in supporting employees diagnosed with cancer, as SMEs do not encounter employees diagnosed with cancer on a regular basis. On the other hand, the small company size may also be seen as an advantage, as it could enhance social support and flexible modifications [27]. Finally, SME’s might fear closing down as a result of an employee diagnosed with cancer [28], lack experience in the management of employees diagnosed with cancer, and have both limited access to occupational health as well as limited support by experienced Human Resource Department [29]. As such, further investigation is required to develop and implement tools and interventions that fit the needs and requirements of SMEs. Third, insights in the structure of relationships among the numerous stakeholders, including the employee diagnosed with cancer, employers, colleagues, treating physicians, and occupational physicians, as well as their perspectives and interests could improve open and effective communication. Effective collaboration between the stakeholders is expected to be a key factor for successful implementation of any intervention that aims to improve RTW of employees diagnosed with cancer [30]. Finally, an essential part of the abovementioned proposed studies should also include the dissemination of results into practice and assess possible facilitators and barriers for implementation of interventions and support tools as it appeared that implementing RTW interventions for cancer survivors is challenging [31, 32].

In conclusion, research in the field of cancer and work has showed us the importance of the employer for work participation of employees diagnosed with cancer. A starting point has been made by studying what employers need in order to support their employee with cancer [20]. However, we currently know little about which aspects of employer support actually have a positive impact on the ability of employees diagnosed with cancer to retain at work or RTW. In addition, there is a lack of interventions and tools which support employers in their management of employees diagnosed with cancer. Future research should focus on which aspects of employer support significantly affect employees diagnosed with cancer to retain at work or RTW and on the development and evaluation of helpful tools and interventions for employers. Research on the evaluation of such tools and interventions should take into account how their results be disseminated to employers to facilitate implementation on a large scale. The inclusion of employer support into the workplace can help employees diagnosed with cancer with work retention and RTW, which is an important aspect of their quality of life and benefits the society at large.
